# Flux-pinning behaviors and mechanism according to dopant level in (Fe, Ti) particle-doped $\text {MgB}_2$ superconductor

**DOI:** 10.1038/s41598-021-89801-4

**Published:** 2021-05-19

**Authors:** H. B. Lee, G. C. Kim, Young Jin Shon, Dongjin Kim, Y. C. Kim

**Affiliations:** Department of Physics, Pusan National University, Busan, 46241 South Korea

**Keywords:** Physics, Condensed-matter physics, Superconducting properties and materials

## Abstract

We have studied flux-pinning effects of $\text {MgB}_2$ superconductor by doping (Fe, Ti) particles of which radius is 163 nm on average. 5 wt.% (Fe, Ti) doped $\text {MgB}_2$ among the specimens showed the best field dependence of magnetization and 25 wt.% one did the worst at 5 K. The difference of field dependence of magnetization of the two specimens increased as temperature increased. Here we show experimental results of (Fe, Ti) particle-doped $\text {MgB}_2$ specimens according to dopant level and the causes of the behaviors. Flux-pinning effect of volume defects-doped superconductor was modeled in ideal state and relative equations were derived. During the study, we had to divide M-H curve of volume defect-dominating superconductor as three discreet regions for analyzing flux-pinning effects, which are diamagnetic increase region after $\text {H}_{c1}$, $\Delta \text {H}=\Delta \text {B}$ region, and diamagnetic decrease region. As a result, flux-pinning effects of volume defects decreased as dopant level increased over the optimal dopant level, which was caused by decrease of flux-pinning limit of a volume defect. And similar behaviors are obtained as dopant level decreased below the optimal dopant level, which was caused by the decreased number of volume defects. Comparing the model with experimental results, deviations increased as dopant level increased over the optimal dopant level, whereas the two was well matched on less dopant level. The behavior is considered to be caused by the segregation of the volume defects. On the other hand, the cause that diamagnetic properties of over-doped $\text {MgB}_2$ specimens dramatically decreased as temperature increased was the double decreases of flux-pinning limit of a volume defect and the segregation effect, which are caused by over-doping and temperature increase.

## Introduction

We have studied flux-pinning effects of $\text {MgB}_2$ superconductor by doping (Fe, Ti) particles of which radius is 163 nm on average. Investigating field dependences of magnetization (M-H curves) of the doped $\text {MgB}_2$ specimens, 5 wt.% doped specimen showed the best M-H curve and M-H curves of other doped specimen became poorer as dopant level increases or decreases. The behavior means that there was the optimal dopant level for the best M-H curve when $\text {MgB}_2$ were doped with the (Fe, Ti) particles. Although the behaviors might be interesting enough, the exact mechanism has not been revealed.

On the other hand, there were several reports that diamagnetic property and critical current ($\text {J}_c$) were changed according to dopant level^1–5^. However, detailed mechanism and the cause have been not shown. According to our studies, one thing to note is that the optimal concentration of defects for the best performance depended on the state of pinning sites that defects produced^6–8^. For example, flux-pinning effects of defects in superconductor would depend on which types, what sizes, and how many.

**Figure 1:**
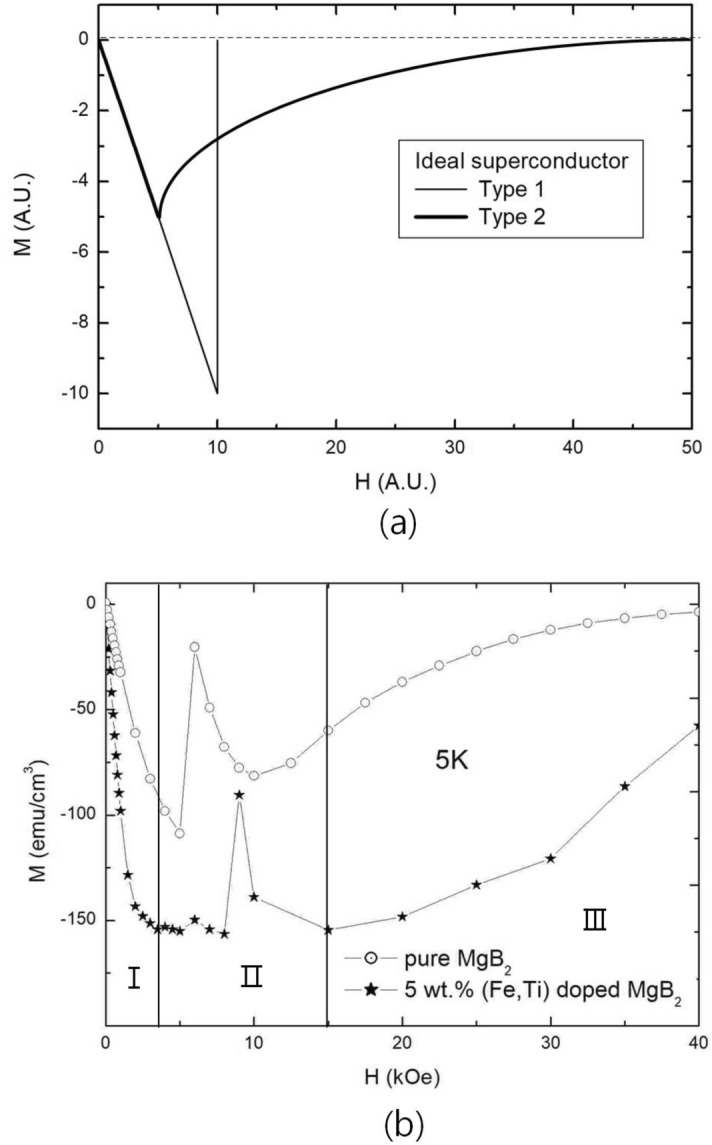
Field dependences of magnetizations (M-H curves) of superconductors (**a**): M-H curves of ideal type I and type II superconductors (**b**): M-H curve of 5 wt.% (Fe, Ti) particle-doped $\text {MgB}_2$ at 5 K, which is volume defect-dominating superconductor. It can be divided as three discreet region, which is diamagnetic increase region (Region I), $\Delta \text {H}=\Delta \text {B}$ region (Region II), diamagnetic decrease region (Region III). M-H curve of pure $\text {MgB}_2$ at 5 K is used as a reference.

The conventional theory for ideal type II superconductor represented two critical fields which are lower critical field ($\text {H}_{c1}$) and upper critical field ($\text {H}_{c2}$) in M-H curve as shown Fig. 1a^9,10^. However, M-H curves of current specimens, which are one of volume defect-dominating superconductors, showed quite different behavior from that of ideal superconductor^11–16^. Thus, we have to explain the M-H curves by dividing them into three discreet regions as shown Fig. 1b, which were developed by our studies^7,8^. The first is the diamagnetic increase region (Region I), the second is $\Delta \text {H}=\Delta \text {B}$ region (Region II) which is the region that increased magnetic field is the same as increasing magnetic induction, and the third is the diamagnetic decrease region (Region III). The cause of the distinction is that M-H curves of real superconductors are heavily influenced by flux-pinning phenomena of defects and each region has different flux-pinning mechanism.

Regarding the region of diamagnetic increase after $\text {H}_{c1}$ (Region I), pinned fluxes at a volume defect are picked out (pick-out depinning) from the defect when F$_{pickout}$ is larger that F$_{pinning}$. The basis of diamagnetic increase after $\text {H}_{c1}$ is that the magnetic fluxes have penetrated into the superconductor after $\text {H}_{c1}$ are pinned at volume defects. Thus, they would be another barrier to prevent the fluxes penetrating into the superconductor if they are pinned at volume defects^8^.

In $\Delta \text {H}=\Delta \text {B}$ region (Region II), a different cause is applied to the movement of pinned fluxes at volume defects. The shortest distance between pinned fluxes at volume defects would determine whether the pinned fluxes have to be pick-out depinned or not. Thus they could be pick-out depinned and move although F$_{pickout}$ is smaller than F$_{pinning}$^17^. Pick-out depinning was named by the behavior of depinning that pinned fluxes are picked out together when they are depinned from the volume defect. The behavior of pinned fluxes in $\Delta \text {H}=\Delta \text {B}$ region are influenced by the nature that neighborhoods of a volume defect would lose superconductivity if the shortest distance between pinned fluxes at the volume defect is the same as that of $\text {H}_{c2}$. Thus, flux-pinning itself is not established in the state and pinned fluxes at a volume defect are pick-out depinned although F$_{pickout}$ is smaller than F$_{pinning}$.

Regarding the diamagnetic decrease region (Region III), it is the region that flux-pinning effect of volume defects decreases. In the region, magnetic fluxes which are not pinned at volume defects increase as applied magnetic field increases and the increase of unpinned fluxes results in a decrease of diamagnetic property of the superconductor by 4$\pi$M = B-H, where M, B and H are magnetization, magnetic induction, and applied magnetic field, respectively. The difference between conventional theory of ideal superconductor after $\text {H}_{c1}$ and Region III of the current representation is the decrease rate of diamagnetic property. The decrease rate was smaller as applied magnetic field increases when flux-pinning effects of volume defects increased (i.e. $\Delta \text {H}=\Delta \text {B}$ region is wider).

In this paper, we modeled flux-pinning effect of volume defects in ideal state according to dopant level in superconductor, and studied the cause of the best field dependence of magnetization of 5 wt.% (Fe, Ti) doped $\text {MgB}_2$ and the cause of gradual decreases of field dependence of magnetization of doped $\text {MgB}_2$ as dopant level decreases or increases from 5 wt.%.

## Results

### Experimental results of field dependences of magnetization for (Fe, Ti) particle-doped $\text {MgB}_2$ specimens

**Figure 2:**
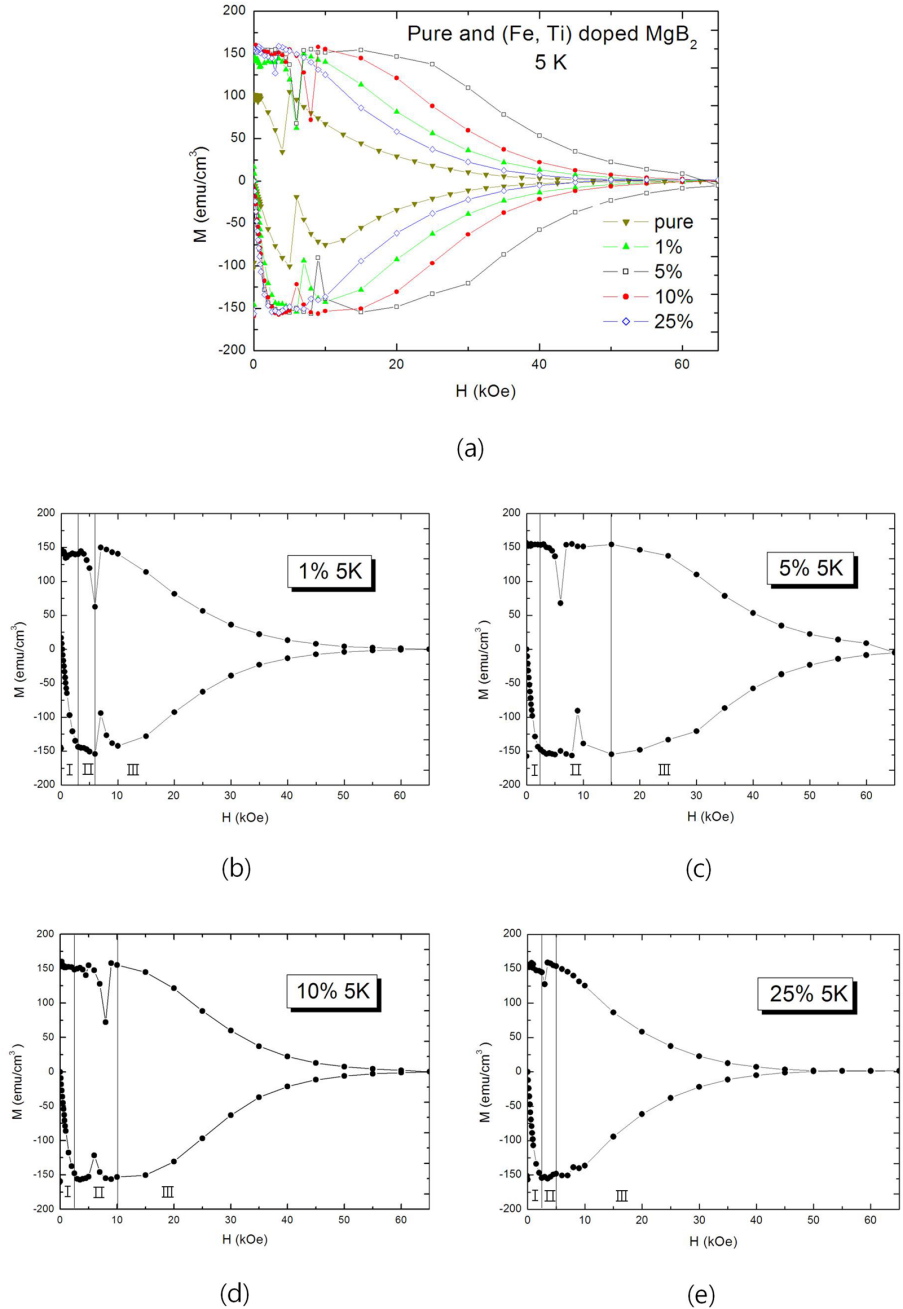
Field dependences of magnetizations (M-H curves) at 5 K of wt.% (Fe, Ti) particle-doped $\text {MgB}_2$ specimens, which was air-cooled. (**a**): M-H curves for comparison. (**b**): M-H curve of 1 wt.% (Fe, Ti) particle-doped $\text {MgB}_2$. (**c**): M-H curve of 5 wt.% (Fe, Ti) particle-doped $\text {MgB}_2$. (**d**): M-H curve of 10 wt.% (Fe, Ti) particle-doped $\text {MgB}_2$. (**e**): M-H curve of 25 wt.% (Fe, Ti) particle-doped $\text {MgB}_2$. Region I and Region II are extended $\Delta \text {H}=\Delta \text {B}$ Region. Decision of a width of $\Delta \text {H}=\Delta \text {B}$ regions of specimens was made by thinking over 1st and 2nd quadrants of M-H curve, and field dependence of magnetization at the same time.

Figure 2a shows M-H curves of pure $\text {MgB}_2$ and (Fe, Ti) particle-doped $\text {MgB}_2$ specimens at 5 K according to dopant level, which are doped with (Fe, Ti) particles of which radius are 163 nm on average. Pure $\text {MgB}_2$ is used as a reference. Inspecting Region I, maximum diamagnetic properties (MDP) of the specimens except pure $\text {MgB}_2$ are almost same. However, they show different widths of Region II, and 5 wt.% doped specimens showed the widest $\Delta \text {H}=\Delta \text {B}$ region. Widths of the region is ordered as follows, 5% > 10% > 1% > 25% > pure. Pure $\text {MgB}_2$ had no $\Delta \text {H}=\Delta \text {B}$ region.

Inspecting Region III in M-H curves as shown in the figure, the decrease rate of diamagnetic property along applied magnetic field was lower as a width of $\Delta \text {H}=\Delta \text {B}$ region was wider. After 5 wt.% doped specimen showed the lowest decrease rate of diamagnetic property along applied magnetic field, decrease rates of other doped specimens increase as dopant level increases from 5 wt.%. And less doped specimens than 5 wt.% also showed same behaviors as dopant level decreases from 5 wt.%.

**Figure 3:**
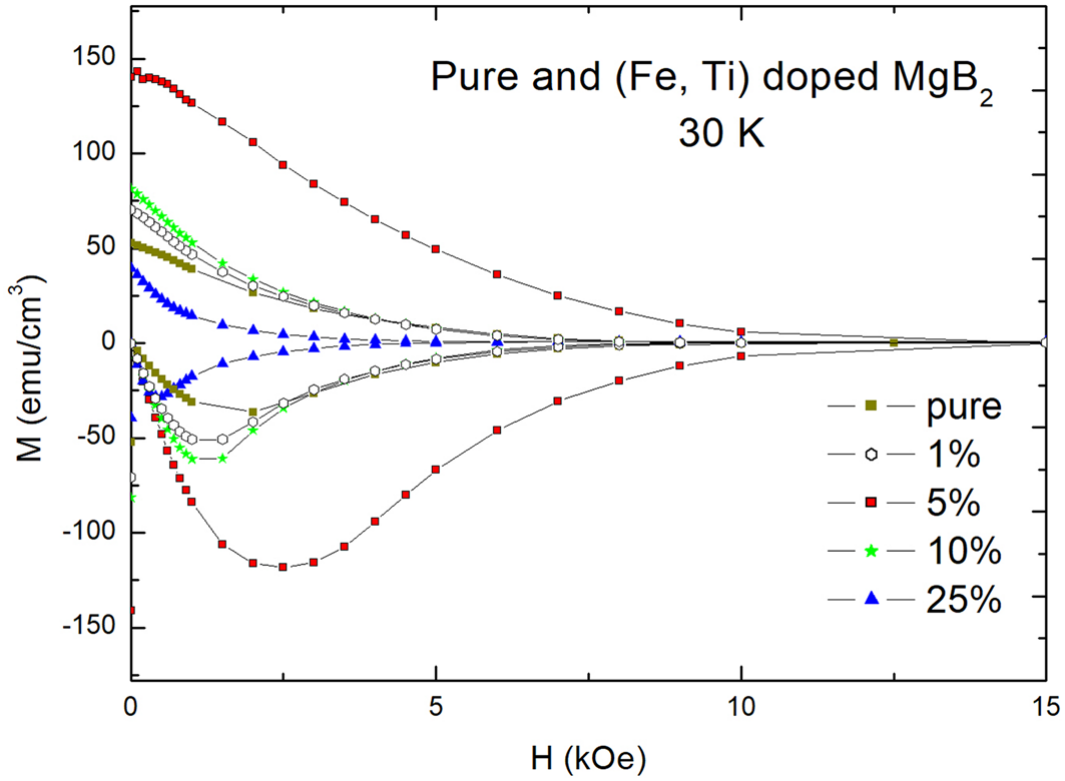
Field dependences of magnetizations (M-H curves) of wt.% (Fe, Ti) particle-doped $\text {MgB}_2$ at 30 K, which were air-cooled.

M-H curves of doped $\text {MgB}_2$ specimens at 30 K are shown in Fig. 3, which is results of the same specimens as shown in Fig. 2. At the temperature, flux-pinning effects of a superconductor have to be determined by heights of MDP and the degree of diamagnetic decrease because $\Delta \text {H}=\Delta \text {B}$ region completely disappeared at 30 K. The specimen showing the best maximum diamagnetic property at 30 K is still 5 wt.% specimen, which had widest $\Delta \text {H}=\Delta \text {B}$ region at 5 K. However, other specimens have some changes on M-H curves. The MDP of 10 wt.% doped specimen is the next, which is same order when compared with a width of $\Delta \text {H}=\Delta \text {B}$ region at 5 K, but it is much closer to that of 1 wt.% doped specimen.

The figure also shows that a difference of diamagnetic decrease between 5 wt.% doped specimen and other specimens at 30 K much increased when compared with those at 5 K. A noting thing is that 25 wt.% doped specimen showed much more diamagnetic decrease along applied magnetic field compared with that of pure $\text {MgB}_2$ at 30 K, which is reversed results at 5 K. From the considerations, it is understood that a degree of diamagnetic decrease at 30 K increases as dopant level increases from 5 wt.% doped specimen.

Considering the experimental results, we have to study mechanisms that could not be explained by conventional theory of superconductivity. The first is the reason that 5 wt.% specimen at 5 K showed the optimal flux-pinning effects. The second is the reason that gradual decrease of a width of $\Delta \text {H}=\Delta \text {B}$ region occurred as the amount of dopant level increases or decreases from optimal dopant level. The third is the reason that a difference of diamagnetic decrease between the optimal flux-pinning specimen and other doped specimens significantly increase when the temperature increases at 30 K. And the fourth is the reason that superconducting properties of doped specimens become much poorer at 30 K as dopant level increases.

### A representation of flux-pinning effect in ideal doped state

**Figure 4:**
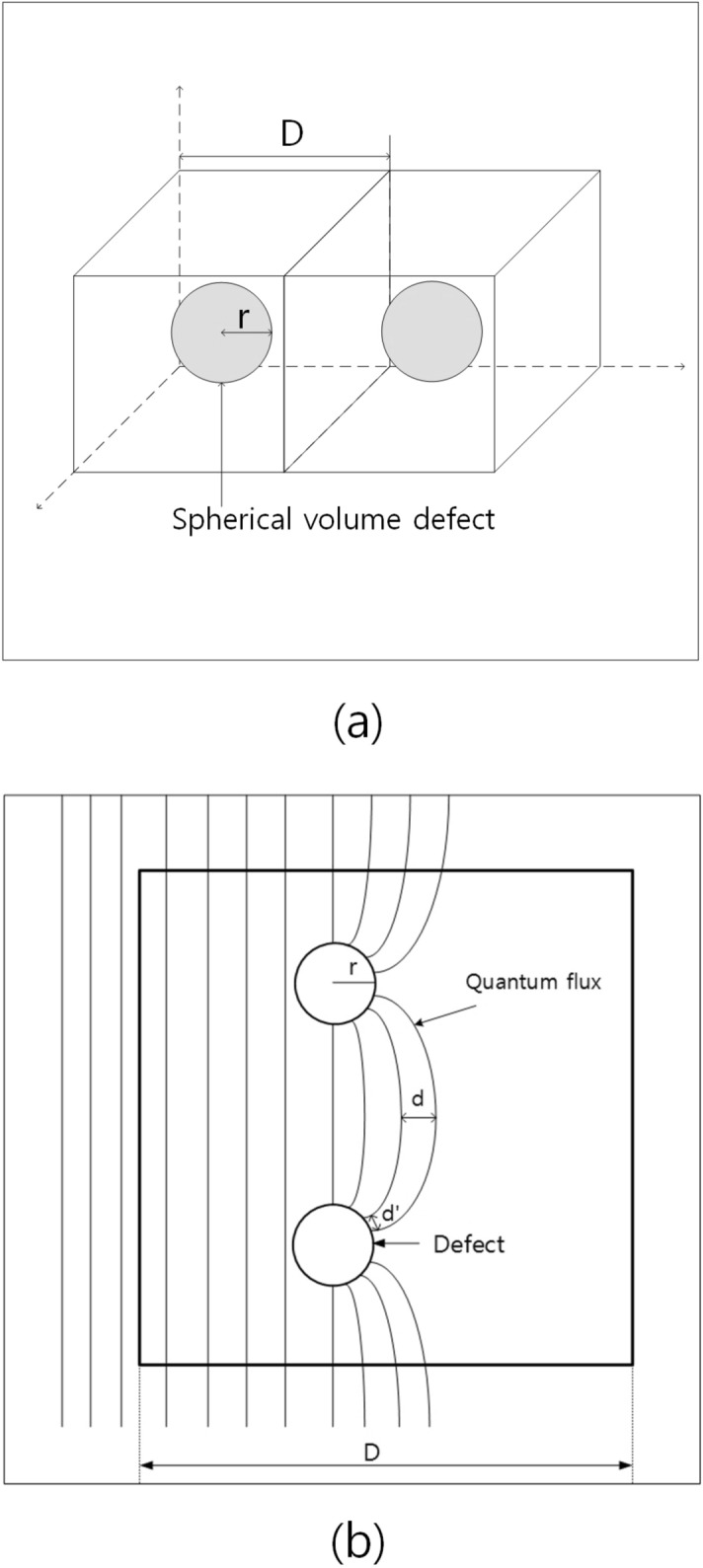
(**a**) Schematic representation of doped volume defects in $\text {MgB}_2$ base. (**b**) Schematic representation of pinned fluxes at volume defects.

Figure 4a shows a schematic representation of volume defect-doped superconductor. It is assumed that spherical volume defects of which radius is *r* are doped in cubic superconductor of which a length is *D*. Figure 4b shows a schematic representation which is several quantum fluxes that are pinned together at volume defects. The shortest distance between pinned fluxes is $d'$ and the widest one is *d*. Generally, the number of quantum fluxes (n$^2$) which can be pinned at a spherical volume defect are calculated as follows.

1 $$\begin{aligned} n^2 = \frac{\pi r^2}{d'^2} \end{aligned}$$

where *r* and $d'$ are radius of volume defect and the shortest distance between quantum fluxes pinned at the volume defect of which radius is *r* when pinned fluxes have square structure, respectively^8^. If $d'^2$ is 2$\pi \xi ^2$ ($\xi$ is coherence length of the superconductor), a volume defect pin the maximum flux quanta, which is flux-pinning limit of a volume defect.

On the other hand, average *d*, which is average value of widest distance between pinned fluxes is

2 $$\begin{aligned} \bar{d} = \frac{D-r}{n} \end{aligned}$$

where *d*, *D*, *r*, and *n* are the widest distance between pinned fluxes, a distance between volume defects, radius of volume defect, and the number of quantum fluxes pinned at volume defects in one dimension (1 D) in the state that they are arrayed in a row to the next volume defect as shown in Fig. 4b.

On the other hand, concerning the widest distance (*d*) between pinned fluxes, the minimal distance is required for flux-pinning effect of volume defects. The reasons are as follows. The first is that forefront fluxes among pinned fluxes at volume defects have more tension, thus the foremost *d* among the pinned fluxes (Fig. 4b) must be shorter than average *d*. And the second is that pinned fluxes are affected by depinnings of other part of fluxes from neighborhood volume defects because pinned fluxes are interconnected from a volume defect to the next. Thus, pinned fluxes are vibrating if they are not depinned when other part of fluxes are depinned. The third is that they are affected by heat caused by depinning of other fluxes and other part of fluxes. Therefore, they cannot maintain flux-pinning state if the distance between pinned fluxes are shorter than the minimal distance, which must be much wider than that of $\text {H}_{c2}$.

The quantum fluxes pinned at volume defects would be divided into two parts, which are volume defect part and superconducting part as shown in Fig. 4b. Since quantum fluxes of volume defect part are fixed on the volume defects, they can withstand until the distance between them is same as that of $\text {H}_{c2}$ However, quantum fluxes of superconducting part are less arrested on the volume defects because they are elastic and away from” the volume defects. Therefore, they are highly affected by other fluxes movements and movements of other parts of quantum fluxes, which are called “the effect of environments”, because a quantum flux is simultaneously pinned at approximately 8000 volume defects a unit length (cm) in 5 wt.% (Fe, Ti) particle-doped $\text {MgB}_2$.

Quantum state of magnetic fluxes would be maintained by the repulsive force generated by superconducting eddy currents^9^. If quantum fluxes failed to be separated by the effect of environments, the superconductivity of the part would disappear at the moment that the distance between fluxes is shorter than that of $\text {H}_{c2}$. The electrons that generate the magnetic quantum flux would be not superconducting electrons anymore, which were Cooper pair. In order to generate magnetic fluxes by normal electrons, it is certain that many electrons have to participate.

Therefore, the heat caused by electric resistance would happen. If the generated heat was small, it does not affect neighborhoods of quantum fluxes to combine, thus they would retain their superconductivity again. However, if it was large enough, it would affect the neighborhoods of quantum fluxes to combine because coherence length of a superconductor increases as temperature increases. Thus, the heat would propagate around it and causes other quantum fluxes to combine into one, which means that they are not a quantum flux anymore. At last, the entire pinned fluxes at volume defect become non-superconducting state. Thus, flux-pinning effects would disappear, and the pinned fluxes naturally are pick-out depinned from the defect. Therefore, the minimal distance (*d*) between pinned fluxes at volume defects must be required to maintain superconductivity of pinned fluxes at a volume defect.

On the other hand, optimal dopant level which shows maximum flux-pinning effect, is defined as the state that flux-pinning limit of a volume defect does not decrease because the minimal distance is maintained although a lot of number of volume defects are doped. Therefore, flux-pinning limit of a volume defects at over-dopant level is

3 $$\begin{aligned} n_{ov}^2 = n_o^2 \left(\frac{D_{ov} - r}{D_o - r}\right)^2 \end{aligned}$$

where $n_o^2$, $D_o$, and $D_{ov}$ are flux-pinning limit of a volume defect at optimal dopant level at 0 K, the distance between volume defects at optimal dopant level, and a distance between volume defects at over-dopant level, respectively. It was assumed that pinned fluxes at a volume are arrayed in square form, thus they are vertically equal.

And a rate of increased flux-pinning sites by over-doping is

4 $$\begin{aligned} R = \frac{m_{ov}^2}{m_o^2} \end{aligned}$$

where $m_o^3$ is the number of volume defects at optimal dopant level and $m_{ov}^3$ is the number of volume defects at over-dopant level. The equation was described because single flux quantum is pinned on volume defects along an axis. Therefore, total flux-pinning effects of volume defects at over-dopant level, which is expressed as an extended width of $\Delta \text {H}=\Delta \text {B}$ region (the entire field that the mechanism of $\Delta \text {H}=\Delta \text {B}$ region is applied, which is from $\text {H}_{c1}$ to the end field of $\Delta \text {H}=\Delta \text {B}$ region) is

5 $$\begin{aligned} W_{\Delta H= \Delta B, ov}=\frac{n_{ov}^2}{n_o^2}\frac{m_{ov}^2}{m_o^2} W_{\Delta H= \Delta B, o} \end{aligned}$$

where $W_{\Delta H= \Delta B, ov}$ and $W_{\Delta H= \Delta B, o}$ are an extended width of $\Delta \text {H}=\Delta \text {B}$ region of over-dopant level and that of optimal dopant level at 0 K, respectively.

On the other hand, under the state that volume defects are in less-dopant level, flux-pinning effects of the superconductor depend on the number of volume defects because flux-pinning limit of a volume defect is the same as that of optimal dopant level.

6 $$\begin{aligned} W_{\Delta H= \Delta B, le}=\frac{m_{le}^2}{m_o^2}W_{\Delta H= \Delta B, o} \end{aligned}$$

where $W_{\Delta H= \Delta B, le}$ is an extended width of $\Delta \text {H}=\Delta \text {B}$ region of less-dopant level and $m_{le}^3$ is the number of volume defects at less-dopant level in a superconductor.

Numerically, 5 wt.% (Fe, Ti) particles in $\text {MgB}_2$ that is optimal dopant level corresponds to approximately 2.0 vol.% and *D* is 5.94*r*. On the other hand, 25 wt.% (Fe, Ti) particles in $\text {MgB}_2$ corresponds to approximately 10 vol.% and *D* is 3.47*r*. A spherical volume defect of 163 nm radius in 5 wt.% (Fe, Ti) particles-doped $\text {MgB}_2$ can pin 51$^2$ flux quanta when $\text {H}_{c2}$ is 65.4 Tesla (T) at 0 K^8^. Assuming that 51 quantum fluxes are arrayed in a row to the next volume defect, $\bar{d}_{5 wt\%}$ is 15.8 nm.

Table 1 shows various properties of (Fe, Ti)-doped $\text {MgB}_2$ specimens according to dopant level. $\bar{d}_{25 wt\%}$ is 7.89 nm for 25 wt.% (Fe, Ti)-doped $\text {MgB}_2$ if 51$^2$ flux quanta are pinned at 163 nm radius volume defect. Superconducting state would be destroyed if *d* is less than 5.63 nm when $\text {H}_{c2}$ is 65.4 T at 0 K. Since the front *d* in pinned fluxes would be shorter than $\bar{d}$ and the effect of environments would affect *d*, it is certain that 163 nm radius volume defect cannot pin 51$^2$ flux quanta in dynamic state.

**Table 1:** Various properties of (Fe, Ti) doped $\text {MgB}_2$ superconductors according to weight percentage of (Fe, Ti) particles, of which average radius is 163 nm.

Weight percentage	1	5	10	25
Volume percentage	0.4	2	4	10
The number of volume defects in cm$^3$	4680$^3$	8000$^3$	10080$^3$	13680$^3$
The distance (L$'$, r = 0.163 $\upmu$m) between volume defect	10.15r	5.94r	4.71r	3.47r
(Ideal state, $\upmu$m)	1.65	0.97	0.68	0.57
The distance (L) between volume defect	85.9r	29.4r	18.5r	10.1r
(Closed packed state, $\upmu$m)	14.0	4.79	3.02	1.64
Calculated flux-pinning effects (width of $\Delta \text {H}=\Delta \text {B}$ region)	0.37	1	0.91	0.75
Experimental results (width of $\Delta \text {H}=\Delta \text {B}$ region)	0.4	1	0.67	0.33
Differences between the calculated and experimental results	0.03	0	0.24	0.42
Flux-pinning limit of a volume defect	51$^2$	51$^2$	38$^2$	26$^2$

L$'$ and L are the distance between defects in ideal state and closed packed state, respectively. Closed packed state of volume defects is the state that all of fluxes penetrated are pinned at volume defects ($2r\times m_{cps}$ = 1, where $m_{cps}$ is the number of volume defects in close packed state^7^. Widths of $\Delta \text {H}=\Delta \text {B}$ region of specimens are compared with that of 5 wt.% (Fe, Ti) doped $\text {MgB}_2$ which is set as a unit.

5 wt.% doped specimen showed the best flux-pinning effect, and of which $\bar{d}_{5 wt\%}$ is 15.8 nm as mentioned. The number of pinned fluxes ($n^2$) at 163 nm radius defect would be approximately 26$^2$ in 25 wt.% doped specimen if $\bar{d}_{5 wt\%}$ is set as the minimal distance. The calculation means that 163 nm radius volume defect of 25 wt.% doped specimen can pin up to only 26$^2$ flux quanta. Therefore, it is considered that the flux-pinning effect of a volume defect in 25 wt.% doped specimen decreases to 0.26 (26$^2$/51$^2$) by Eq. (3).

On the other hand, increased flux-pinning sites of 25 wt.% doped specimen is 2.92 (13,680$^2$/8000$^2$) by Eq. (4) because single flux quantum is pinned on 13,680 volume defects per unit length (cm) along an axis. Thus, actual rate of flux-pinning effect by increased volume defects for 25 wt.% doped specimen are 0.75 (0.26 × 2.92) by Eq. (5). Table 1 also shows actual flux-pinning effect of increased volume defects for 10 wt.% doped specimen, which is 0.91. On the other hand, decreased flux-pinning effect of 1 wt.% specimen is 0.37 (4860$^2$/8000$^2$) by Eq. (6).

Comparing theoretical values with the experimental results as shown in Table 1, extended widths of $\Delta \text {H}=\Delta \text {B}$ region of 5 wt.%, 10 wt.%, 25 wt.%, and 1 wt.% specimens are experimentally 1.5 Tesla (T), 1.0 T, 0.5 T, and 0.6 T, respectively, as shown Fig. 3. 1 wt.% specimen shows a good match with theoretical value (0.6 T/1.5 T = 0.4). However, as the dopant level increases from 5 wt.%, a difference between the experimental results and those of calculations increases (10 wt.% specimen: 0.91 – 0.67 = 0.23, 25 wt.% specimen: 0.75 – 0.33 = 0.42). This means that there would be other factors decreasing flux-pinning effect in over-doped specimens.

### The segregation effect of volume defects and temperature dependence of a width of $\Delta \text {H}=\Delta \text {B}$ region at over-dopant level

As the number of doped volume defects increases in a superconductor, average distance between them do not only decrease, but volume defects which are closer than average distance also increase. As discussed earlier, the shorter the distance between volume defects is, the fewer flux-pinning limit of a volume defect is. Furthermore, if the distance between them is shorter than $\sqrt{2\pi }\xi$ of the superconductor, they are recognized as a pinning site. Thus, magnetic fluxes would feel that the volume defects are connected each other in the state. Therefore, the fluxes move more easily along the volume defects. Consequently, the fluxes can quickly penetrate into an inside of the superconductor when applied magnetic field increases because the segregated volume defects have lower flux-pinning limits.

The flux-pinning limit of a volume defect decreases as temperature increases, which is caused by increase of coherence length as temperature increases. Therefore, an extended width of $\Delta \text {H}=\Delta \text {B}$ region of optimal dopant level at a temperature is

7 $$\begin{aligned} W_{\Delta H= \Delta B(T),o} = \frac{n^2_{T}}{n_o^2}W_{\Delta H= \Delta B(0), o} \end{aligned}$$

where $n^2_T$ is flux-pinning limit of a volume defect at a temperature and $W_{\Delta H= \Delta B(0), o}$ is an extended width of $\Delta \text {H}=\Delta \text {B}$ region of optimal dopant level at 0 K.

In the state of over-dopant level, an extended width of $\Delta \text {H}=\Delta \text {B}$ region of at a temperature is

8 $$\begin{aligned} W_{\Delta H= \Delta B(T), ov}= \frac{n_{ov(T)}^2}{n_{ov(0)}^2} W_{\Delta H= \Delta B(0), {ov}} \end{aligned}$$

On the other hand, as temperature increases, over-doped superconductor would have much lower flux-pinning effect because volume defects have two kinds of decreases of flux-pinning limits. One is a decrease of flux-pinning limit of a volume defect by over-doping, and the other is that of temperature increase, the two both are caused by increase of coherence length as temperature increases. Therefore,

9 $$\begin{aligned} W_{\Delta H= \Delta B(T), ov}= \frac{n_{ov(T)}^2}{n_{o(0)}^2}\frac{m_{ov}^2}{m_o^2}W_{\Delta H=\Delta B(0), o} \end{aligned}$$

The last Equation came from Eq. (5). Therefore, flux-pinning effects of a superconductor at over-dopant level ($W_{\Delta H= \Delta B(T), ov}$) decrease dramatically as temperature increases compared with that of optimal dopant level because $n_{ov(T)}^2$ decreases dramatically as temperature increases

It is expected that M-H curves of over-doped specimens would be much poorer than those of optimal and less-doped specimen as temperature increases. Figure 3 shows those behaviors, which are that M-H curve of the 25 wt.% doped $\text {MgB}_2$ showed poorer M-H curve than that of pure $\text {MgB}_2$ at 30 K although the M-H curve of 25 wt.% doped specimen showed better M-H curve than that of pure $\text {MgB}_2$ at 5 K.

### Discussion

We suggested that M-H curves of real Type II superconductors have to be considered as three discreet regions owing to their different causes for the regions as shown Fig. 2b. If there were no defects in a superconductor, which means no flux-pinning effect, the M-H curve would reduce to ideal superconductor by no increasing diamagnetic property in Region I and deleting $\Delta \text {H}=\Delta \text {B}$ region, which is Region II. And M-H curve become three Regions if volume defects are many enough as being explained. When planar defects are dominant in a superconductor, presence of Region II depends on the dominance of planar defects. Region II completely disappears when dominance of planar defects are high, such as HTSC bulks which were made by solid state reaction method because flux quanta move fast along grain boundaries although they have flux-pinning effects^18,19^.

However, Region II partially appears in the state that dominance of planar defects is low such as water-quenched $\text {MgB}_2$ specimen^20^. In addition, volume defects do not only increase diamagnetic property of the superconductor, but also form Region II. The behavior means that $\text {H}_{c2}$ state partially appears in Region II by pinned fluxes at volume defects. The behavior is based on the nature that pinned fluxes at volume defects are pick-out depinned from the volume defect when the shortest distance between pinned fluxes is the same as that at $\text {H}_{c2}$^7^.

**Figure 5:**
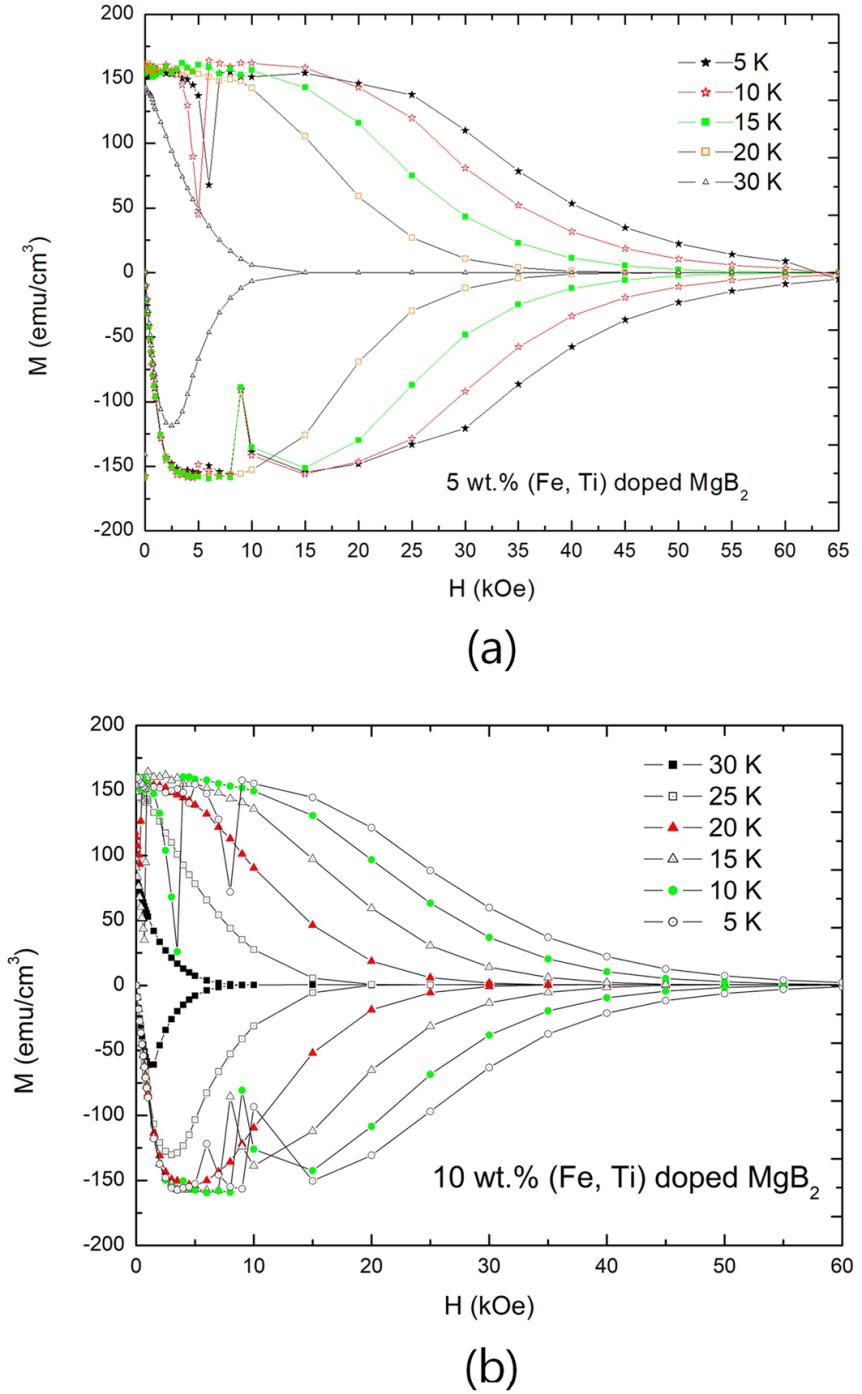
M-H curves of (Fe, Ti) particle-doped $\text {MgB}_2$ superconductors with variation of temperature, which were air-cooled. (**a**) M-H curves of 5 wt.% (Fe, Ti) particle-doped $\text {MgB}_2$ with variation of temperature. (**b**) M-H curves of 10 wt.% (Fe, Ti) particle-doped $\text {MgB}_2$ with variation of temperature.

Region III is the region that flux-pinning effects of the volume defects are weakened as mentioned. Figure 5a,b shows examples of the behavior. They show diamagnetic properties of 5 wt.% and 10 wt.% (Fe, Ti)-doped $\text {MgB}_2$ along applied magnetic field at various temperatures, respectively. Generally, the better flux-pinning state of the volume defects are, the wider $\Delta \text {H}=\Delta \text {B}$ region is formed in Region II. Thus, the specimens have shown that wider $\Delta \text {H}=\Delta \text {B}$ region induced smaller decreases of diamagnetic properties in Region III as applied magnetic field increases. Therefore, it is determined that Region III also affected by flux-pinning effects of volume defects.

## Conclusion

We studied flux-pinning effects of (Fe, Ti) particle-doped $\text {MgB}_2$ specimens according to dopant levels of (Fe, Ti) particles, of which radius is 163 nm on average. M-H curves of the specimens were explained as three discreet regions for flux-pinning effects of volume defects, which are diamagnetic increase region after $\text {H}_{c1}$ (Region I), $\Delta \text {H}=\Delta \text {B}$ region (Region II), and diamagnetic decrease region (Region III). Flux-pinning effect of volume defects-doped superconductor was modeled in an ideal state that the numbers of doped volume defects are varied from the optimal dopant level and relative equations were derived. We focused a width of $\Delta \text {H}=\Delta \text {B}$ region in comparing theoretical values with experimental results because a degree of diamagnetic decrease is inversely proportional to a width of $\Delta \text {H}=\Delta \text {B}$ region and max-diamagnetic properties of the doped specimens were almost same.

Results of experiments represented that 5 wt.% (Fe, Ti)-doped $\text {MgB}_2$ showed widest $\Delta \text {H}=\Delta \text {B}$ region and smallest diamagnetic decrease in diamagnetic decrease region among doped specimens, and widths of $\Delta \text {H}=\Delta \text {B}$ region gradually became shorter as dopant level increases or decreases from 5 wt.%. The causes of the behavior were decreases of flux-pinning limit of a volume defect for over-dopant level and a decrease of the number of volume defects for less-dopant level. Comparing experimental results with theory, the two well matched at less-dopant level than optimal dopant level, but the difference between the two increased as dopant level increases from optimal dopant level. On the other hand, widths of $\Delta \text {H}=\Delta \text {B}$ region became much shorter as temperature increases as dopant level increased, which means decreases of flux-pinning limit of a volume defect. Inspecting the cause, the behavior was revealed as double decreases of flux-pinning limit of a volume defect which are caused by over-doping and temperature increase, and the segregation effect.

## Method

(Fe, Ti) particle-doped $\text {MgB}_{2}$ specimens were synthesized using the non-special atmosphere synthesis (NAS) method^21^. The starting materials were Mg (99.9% powder) and B (96.6% amorphous powder) and (Fe, Ti) particles. Mixed Mg and B stoichiometry, and (Fe, Ti) particles were added by weight. They were finely ground and pressed into 10 mm diameter pellets. (Fe, Ti) particles were ball-milled for several days, and average radius of (Fe, Ti) particles was about 0.163 $\upmu$m. On the other hand, an 8 m-long stainless- steel (304) tube was cut into 10 cm pieces. One side of the 10 cm-long tube was forged and welded. The pellets and excess Mg were placed in the stainless-steel tube. The pellets were annealed at 300 °C for 1 h to make them hard before inserting them into the stainless-steel tube. The other side of the stainless-steel tube was also forged. High-purity Ar gas was put into the stainless-steel tube, and which was then welded. Specimens had been synthesized at 920 °C for 1 hour and cooled in air. Field dependences of magnetization were measured using a MPMS-7 (Quantum Design). During the measurement, sweeping rates of all specimens were made equal for the same flux-penetrating condition.
